# Performance analysis of heterostructure-based topological nanophotonic sensor

**DOI:** 10.1038/s41598-023-46784-8

**Published:** 2023-11-08

**Authors:** Amit Kumar Goyal, Ajay Kumar, Yehia Massoud

**Affiliations:** 1 Innovative Technologies Laboratories (ITL), King Abdullah University of Science and Technology (KAUST), 23955 Thuwal, Saudi Arabia; 2 ECE Department, Jaypee Institute of Information Technology (JIIT), Noida, 201309 India

**Keywords:** Optical sensors, Nanophotonics and plasmonics, Nanophotonics and plasmonics, Surfaces, interfaces and thin films

## Abstract

In this manuscript, a heterostructure-based topological nanophotonic structure is proposed for improved sensing performance. The topological effect is realized by connecting two dissimilar one-dimensional photonic crystal structures having overlapped photonic bandgaps. The structural parameters are optimized to regulate and alter the dispersion characteristics, which results in the opposite Zak phases. This demonstrates a robust topologsical interface state excitation at a 1737 nm operating wavelength. Further, a topological cavity structure having resonance mode at 1659 nm is formed by replacing the interface layers with a defect layer. The mode excitation is confirmed by analyzing the electric field confinement at the interface. The sensing capability of the structure is analytically evaluated by infiltrating different analytes within the cavity. The analytical results demonstrate the device’s average sensitivity of around 774 nm/Refractive index unit (RIU) along with an average high Q-factor and figure of merit of around 5.2 × 10^4^ and 2.6234 × 10^4^ RIU^−1^, respectively. Because of the higher interface mode field confinement, the proposed structure exhibits a 92% higher sensitivity, 98% improved Quality factor, 206% improvement in figure of merit, and 86% higher interface field confinement than conventional Fabry–Perot resonator structures. Thus, the proposed topological cavity structure shows its broad sensing ability (Refractive Index: 1.3–1.6) along with a low-cost, simple fabrication and characterization process, promoting the development of highly sensitive planner nanophotonic devices.

## Introduction

Topological insulators have widely attracted broad interest because of their exciting and unique properties to confine the topologically protected edge states (TES)^1–3^. These TES have been used in various applications because of their robustness against surrounding perturbations^4,5^. They also offer improvement and manipulation of light-matter interactions like electromagnetically induced transparency peaks in multilayer grating structures^6,7^. Thus exhibiting propagation of low scattered and topologically protected edge modes^8,9^. Recently, topological edge properties of the nanophotonic structure have also been explored. It has been demonstrated that optical waveguides^10^, Photonic crystal (PhC) structures^11–13^, Nanoparticles^14^, and acoustic structures^15^ can excite TES. Among the considered structures, one-dimensional photonic crystal structures (1D-PhC) structures are considered promising ones because of their design flexibility at various operating wavelengths, material availability, easy fabrication, and characterization characteristics^16–18^. Additionally, these structures possess the capability to be used as refractive index sensors^19–21^.

The topological states can be excited at the interface of two 1D-PhC structures having overlapping bandgaps and different topological properties or Zak phase^22,23^. Having different Zak phases at a considered operating wavelength leads to the formation of band inversion and, hence, excitation of topological mode^24^. Gao et al.^25^ proposed an interference setup to measure the Zak phase of the 1D-PhC structure and predicted the existence of the TES. This can also be accomplished by calculating the structure's surface impedance related to the Zak phase. Xiao et al.^11^ measured the surface impedance of two 1D-PhC structures to determine the existence of TES at the interface theoretically. Based on these approaches, 1D-PhC-based TES has been used to design a refractive index (RI) sensor with 254.5 nm/RIU sensitivity^26^. The sensitivity is enhanced to a 616 nm/RIU value by incorporating an electro-optical (EO) material between the two 1D-PhC structures^27^. The TES concept in 1D-PhC is further extended to study the plasmonic properties of the device. Lu et al.^28^ investigated the enhancement of plasmonic Tamm states using the topological property of a periodic structure. Further, a graphene-based multichannel absorber is proposed by coupling the topological states with Tamm plasmon polaritons^29^. The authors reported a more than 97% absorption in the incidence angle range of 0° to 50°. Gao et al. used three 1D-PhC structures and a defect layer to excite a Fano resonance using the structure's combined topological property and Fabry–Perot cavity^30^. Recently, in 2022, TES in 1D-PhC, along with three-dimensional Dirac semimetal, is utilized to realize a low threshold optical bistability^31^. Furthermore, a polarization-independent optical biosensor has also been proposed to have a sensitivity of 70°/RIU (60°/RIU) for TE (TM) polarized light^32^. Therefore, 1D-PhC structures possess the capability to have superior topological properties, which can be used to manipulate light-matter interactions and thus show their potential applications in low-concentration analyte detection with the improved figure of merit (FOM). However, in most of the reported TES sensors, the considered refractive index range is very low (1.3–1.4), thus not suitable for wide sensing applications. Moreover, the reported sensitivity and FOM values are also significantly less.

In this paper, the localization of topological interface modes is analyzed for a dielectric-based 1D-PhC heterostructure. Two 1D-PhC structures made of silicon and silicon dioxide (SiO_2_) materials having overlapping bandgaps are considered. The analytical results are verified using the finite element method (FEM) of COMSOL Multiphysics. The parameters are optimized to modify the dispersion characteristics, which results in the opposite topological properties (Zak Phase) at the overlapping bandgap region. This exhibits the excitation of TES for 1737 nm operating wavelength at the interface having very high electric field intensity. The topological cavity is formed by replacing the two interface layers with an aqueous defect layer. The impact of varying defect layer thickness on excited topological interface mode is also studied in detail. This confirms the robustness of TES in the proposed design. Infiltrating the defect layer with an analyte of varying refractive index (1.3–1.6) leads to a redshift in the topological cavity resonance wavelength, which is measured to calculate the device’s sensitivity. The structure performance is also compared with the conventional Fabry–Perot (FP)- cavities having the same design parameters. The proposed structure shows 92% higher sensitivity, 98% improved Quality factor, and 206% improvement in the FOM. Moreover, the TPhC cavity structure exhibits about 86% higher interface field confinement than the conventional FP-cavity structure. Finally, the structural performance is also compared with recently reported values. Therefore, the proposed device provides a high-performance sensor for medical and commercial applications with a simple structure and low cost.

## Theoretical analysis and methods

**Figure 1:**
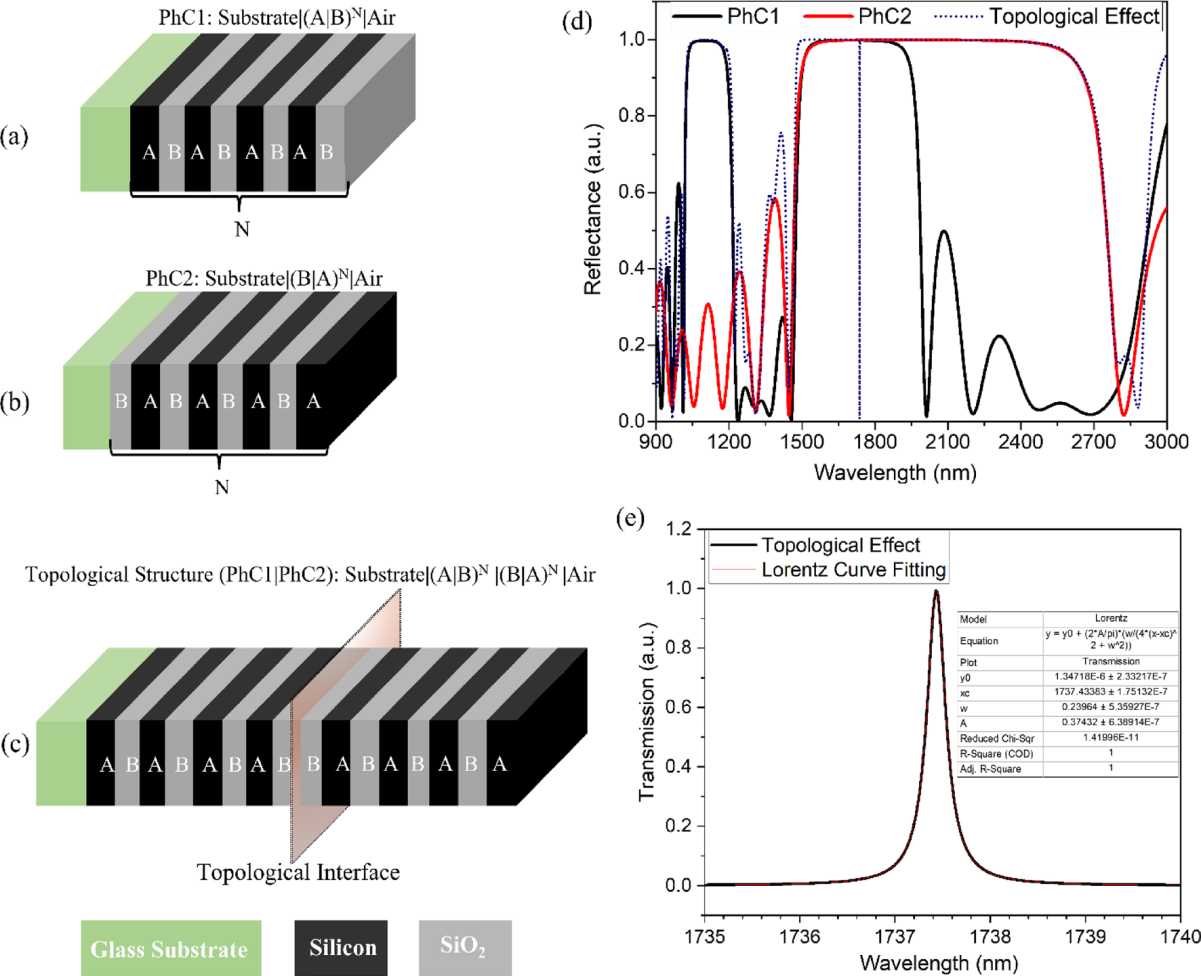
Schematic representation of the 1D-PhC heterostructure (**a**) PhC 1, (**b**) PhC2, (**c**) Combined structure for TES excitation, (**d**) Corresponding reflectance spectrum, and (**e**) Lorentz curve fitting of TES.

The schematic representation of the proposed 1D-PhC heterostructure is shown in Fig. 1. Here, two 1D-PhC structures (PhC1 and PhC2) made of the same material ‘A’ (of Silicon having RI (3.45)) and ‘B’ (of SiO_2_ having RI (1.45)) are considered. The layer thicknesses are optimized to have overlapping bandgaps. The PhC1 comprises four alternate layers (N = 4) of material ‘A’ and ‘B’, thus forming the “Substrate|(A|B)^N^|air” configuration as shown in Fig. 1a. The structure possesses two photonic band gaps (PBG) of width 223 nm (1012 nm-1235 nm) and 554 nm (1455 nm-2009 nm) for considered layer thicknesses D_A1_ and D_B1_ of 380 nm and 250 nm, respectively, as shown by the black curve in Fig. 1d. Similarly, PhC2 also comprises four alternate layers (N = 4) of material ‘B’ and ‘A’, thus forming the “Substrate|(B|A)^N^|air” configuration as shown in Fig. 1b. The structure possesses one wider PBG of width 1379 nm (1445–2824 nm) for considered layer thicknesses D_A2_ and D_B2_ of 140 nm and 320 nm, respectively, as shown by the red curve in Fig. 1d.

The topological states can be excited at the interface of two 1D-PhC structures having overlapping bandgaps and different topological properties or Zak phase. It is clear from Fig. 1d that both structures have an overlapping PBG in the range of 1455–2009 nm; thus, it is possible to excite a TES provided both PhC1 and PhC2 have different topological properties or Zak phase in this overlapping PBG region. The Zak phase generally has quantized phase values of 0 or π and can be calculated from the eigenfrequency band of 1D-PhC as described in Eq. 1^11,33^.

1 $$\theta_{n}^{Zak} = \mathop \smallint \limits_{{{\raise0.7ex\hbox{${ - \pi }$} \!\mathord{\left/ {\vphantom {{ - \pi } \Lambda }}\right.\kern-0pt} \!\lower0.7ex\hbox{$\Lambda $}}}}^{{{\raise0.7ex\hbox{$\pi $} \!\mathord{\left/ {\vphantom {\pi \Lambda }}\right.\kern-0pt} \!\lower0.7ex\hbox{$\Lambda $}}}} \left[ {i\mathop \smallint \limits_{unit cell} dz \varepsilon \left( z \right)u_{n,k}^{*} \left( z \right)\partial_{k} u_{n,k} \left( z \right)} \right]dk$$

Where $k$ is the wave vector, $\varepsilon \left( z \right)$ is the dielectric function, $u_{n,k} \left( z \right)$ is the Bloch electric field eigenfunction of the n^th^ band. The transmission matrix method (TMM) is further used to calculate the $u_{n,k} \left( z \right)$ (i.e. $E_{n,k} \left( z \right) = u_{n,k} \left( z \right) e^{ikz}$). Thereby, calculating the Zak phase for individual PBG provides topological properties. Here, both overlapping PBGs possess different topological properties. Thus, the excitation of TES is expected at the interface of the combined structure of PhC1 and PhC2. The combined structure forming the “Substrate|(A|B)^4^|(B|A)^4^|air” configuration having the topological interface is shown in Fig. 1c, and the corresponding reflectance spectrum is shown in Fig. 1d. The structure possesses excitation of a TES at 1737 nm, having FWHM of around 0.23 nm, as shown by the blue curve in Fig. 1d. Moreover, the exited TES possesses a Lorentzian curve shape, as shown in Fig. 1e. Notably, the TES is excited because of the topological property of the structure and possesses very high transmission (~ 100%) at the interface. Moreover, the excited TES is invariant to the small surrounding perturbation.

**Figure 2:**
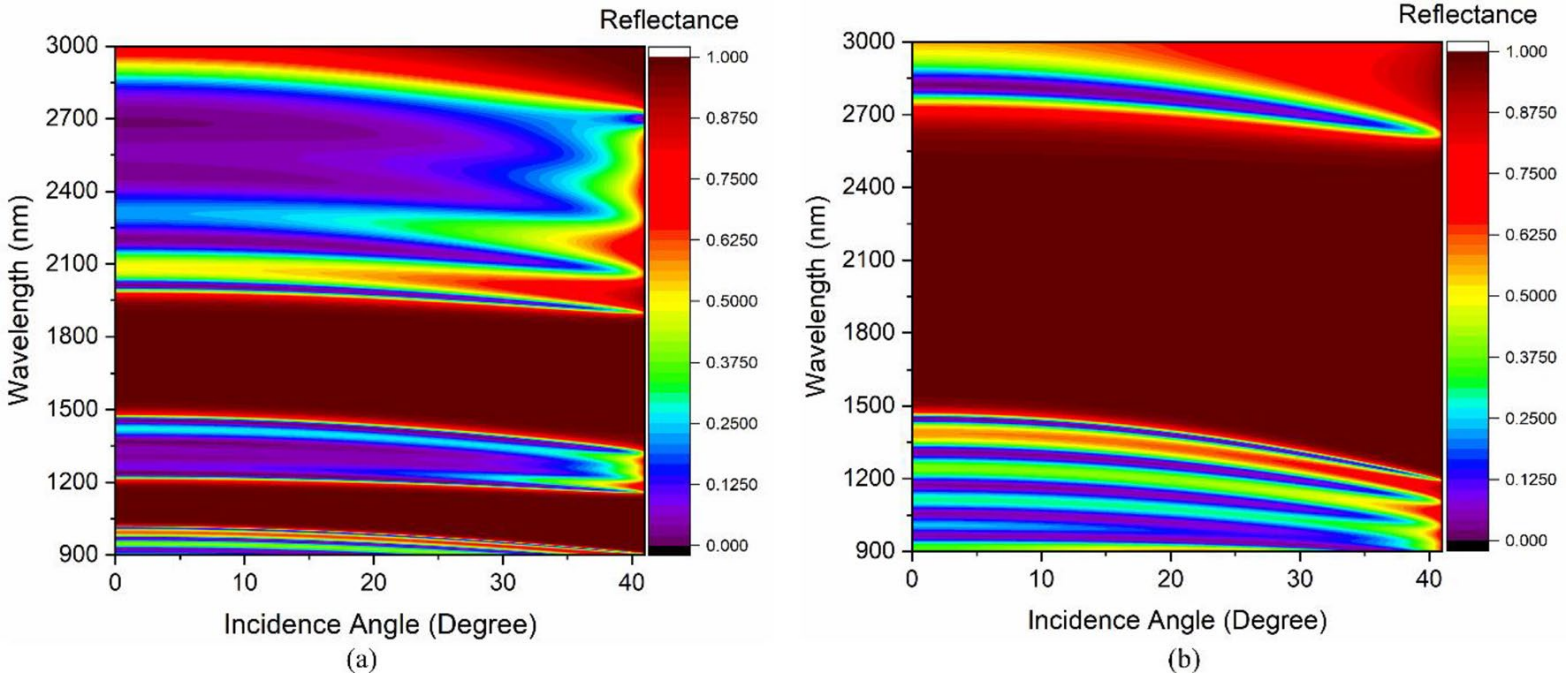
The impact of incidence angle on PBG property of (**a**) PhC1 structure and (**b**) PhC2 structure.

**Figure 3:**
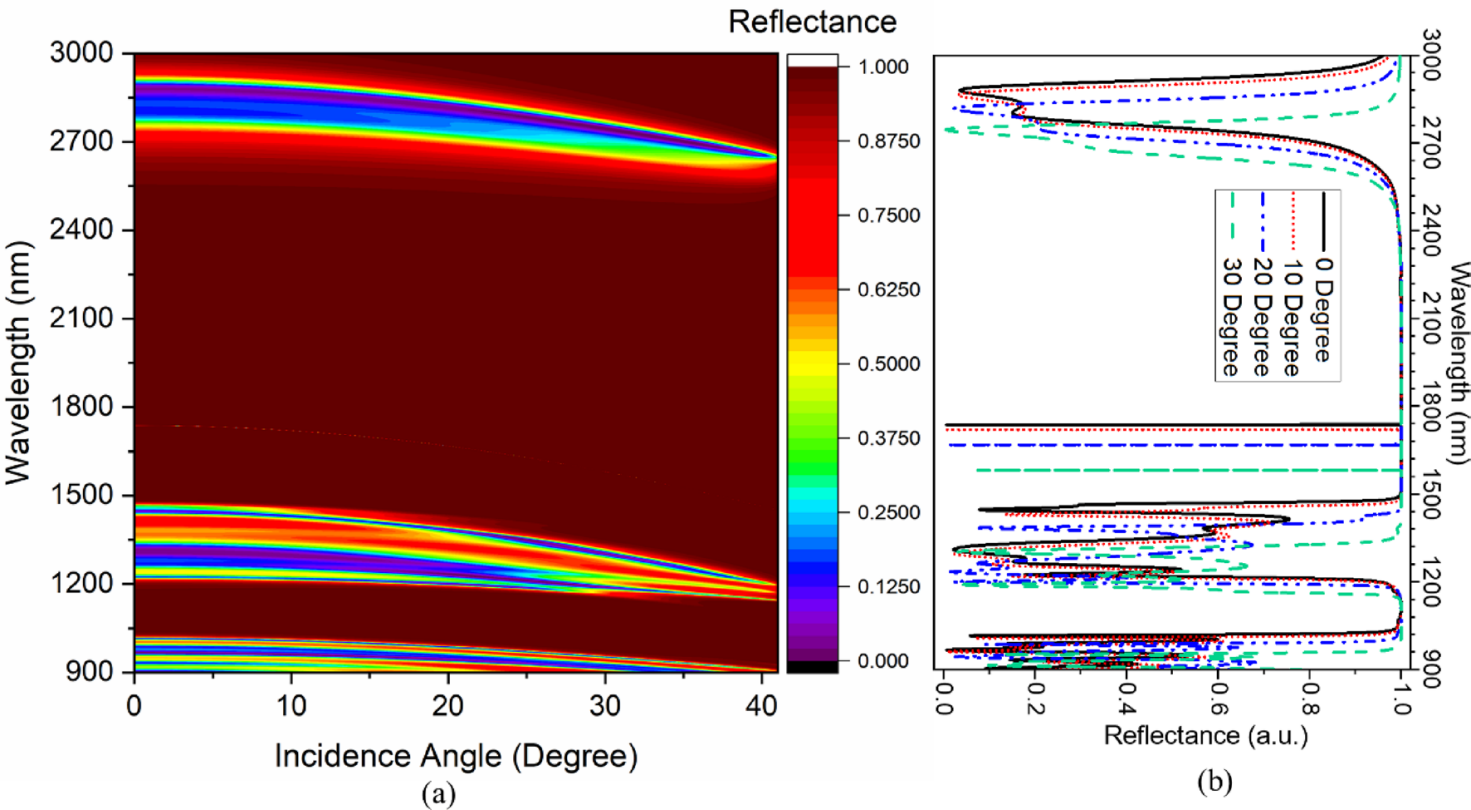
(**a**) Angular dispersion analysis of topological structures, and (**b**) Corresponding reflectance spectrum.

The analysis is further extended to see the impact of incidence angle on the TES excitation characteristics of the devices. Figure 2a represents the impact of the incidence angle on the PBG of PhC1, whereas the effect of the incidence angle on the PBG for PhC2 structure is shown in Fig. 2b. The designs show an excellent overlapping PBG having different topological properties up to 40° incidence angle. Therefore, it exhibits the excitation of TES at the interface of PhC1 and PhC2 and is represented in Fig. 3. Figure 3a illustrates the angular dispersion analysis of the combined structure of Fig. 1c, and the corresponding reflectance spectrum is shown in Fig. 3b. Increasing the incidence angle from 0° to 40° leads to the excitation of TES, which can be confirmed by the appearance of the mode profile in the PBG of Fig. 3a. The structure shows TES excitation at 1737 nm, 1720 nm, 1668 nm, 1581 nm, and 1463 nm operating wavelengths for corresponding incidence angles of 0°, 10°, 20°, 30°, and 40°, respectively, as shown in Fig. 3b. The blue shifting of TES excitation is because of combined Bragg-Snell’s law^34^. Therefore, the topological edge state excitation wavelength can also be tuned by varying the incidence angle.

## Sensing performance analysis

**Figure 4:**
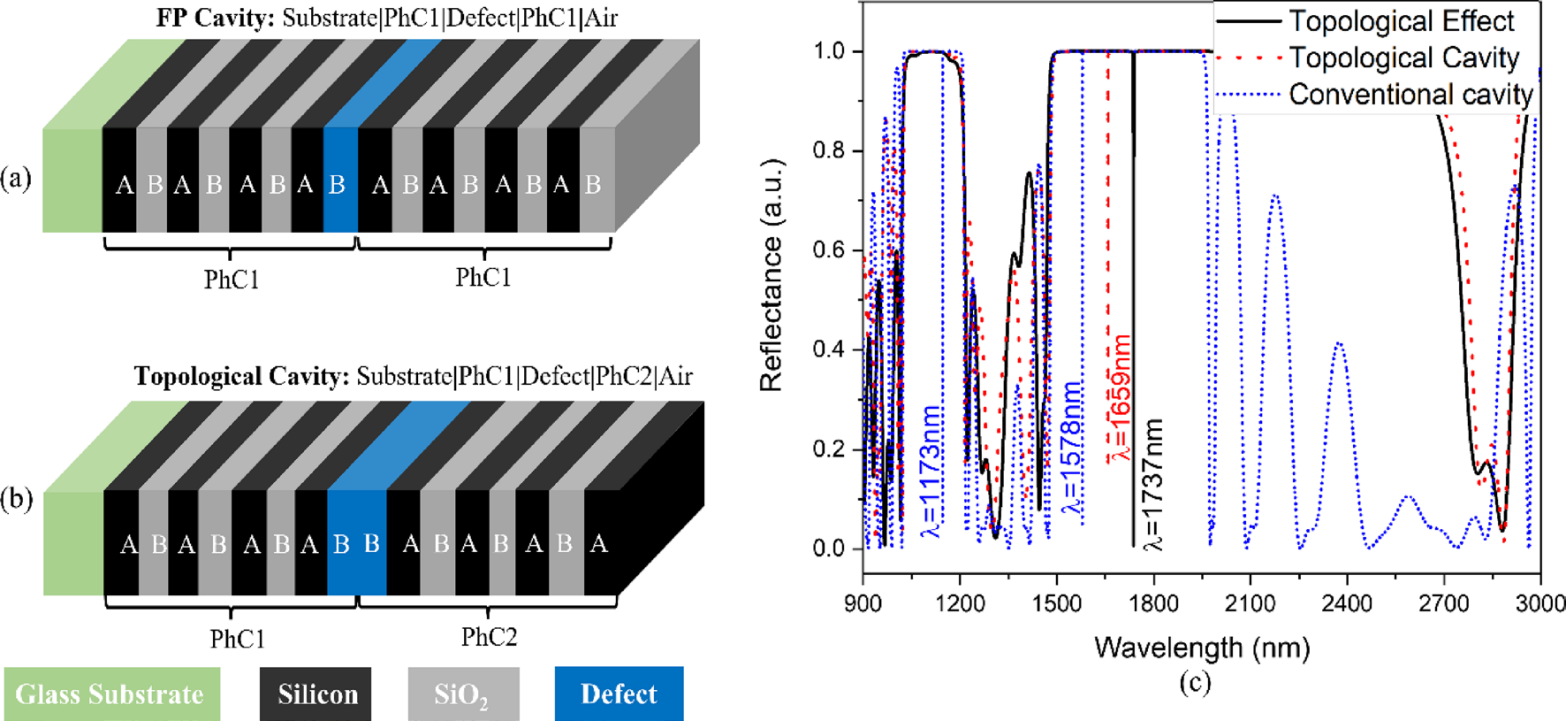
Schematic representation of (**a**) Conventional FP-cavity structures, (**b**) Topological-cavity structure, and (**c**) Corresponding combined reflectance spectrum.

The topological effect can be used to create highly sensitive devices by modulating the interface of PhC1 and PhC2. Figure 4 shows the conventional FP-cavity structure and interface-modulated topological cavity structure. The conventional FP-cavity is designed using two similar PhC1 structures, where the middle low index interface layer is replaced by a defect layer with an aqueous analyte of a refractive index of 1.30. This forms the “Substrate**|**PhC1**|**D**|**PhC1**|**Air” configuration, as shown in Fig. 3a, where the blue color represents the defect layer of thickness Dd (here, Dd = 2D_B1_). Similarly, the topological cavity is designed by replacing two low-index interface layers with a defect layer of an aqueous analyte having a 1.30 refractive index. This forms the “Substrate**|**(A**|**B)^3^**|**A**|**D**|**A**|**(A**|**B)^3^**|**Air” configuration, as shown in Fig. 3b, where the blue color represents the defect layer of thickness Dd (here Dd = D_B1_ + D_B2_). Both structures show very good excitation of a resonating mode within the defect cavity structure. The FP-cavity results in the excitation of the resonating mode at 1578 nm. In contrast, the resonance mode for the topological cavity is excited at a resonance wavelength of 1659 nm, which is shown by the corresponding reflectance spectrum of Fig. 4c.

**Figure 5:**
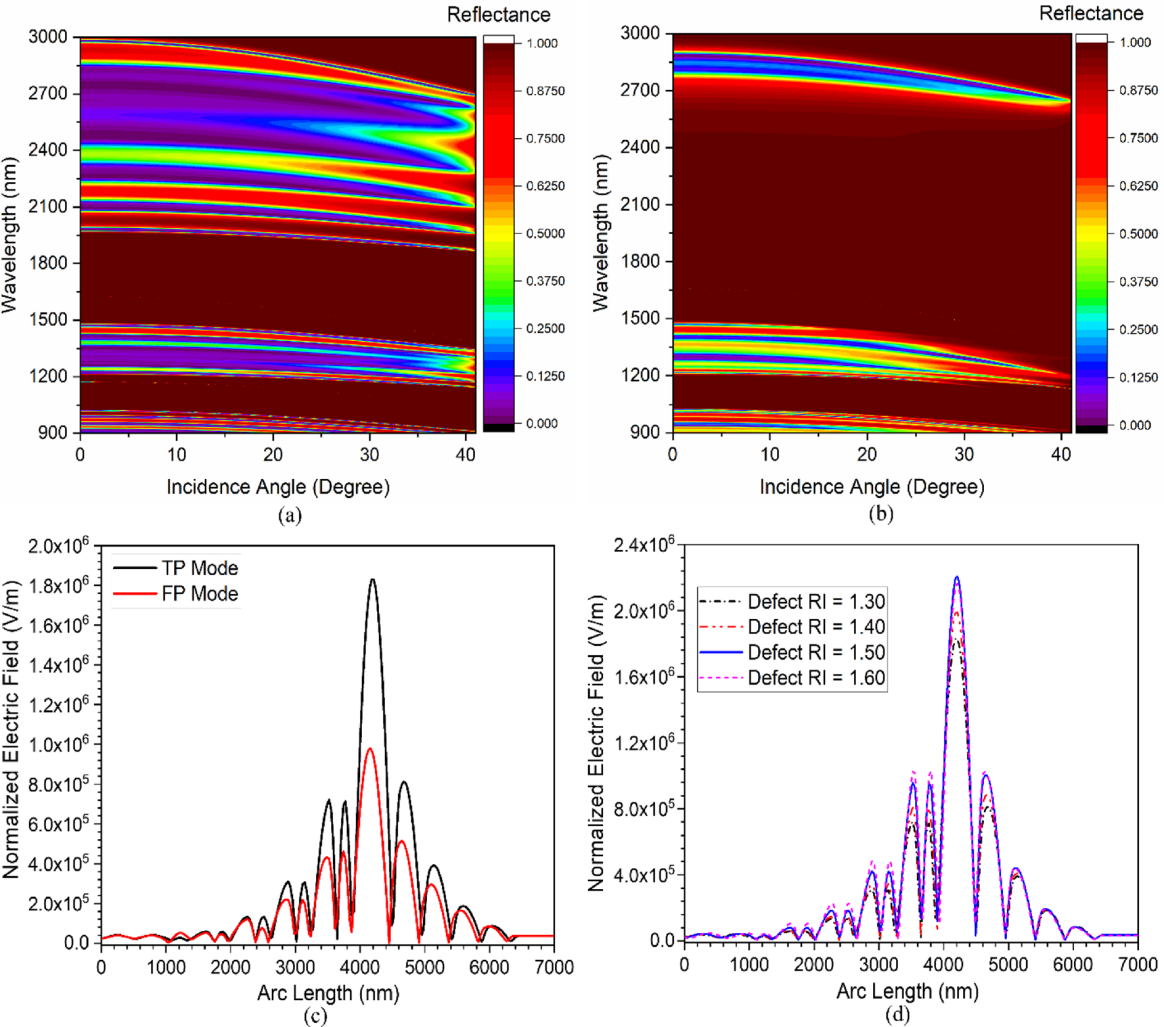
Angular dispersion analysis of (**a**) FP-cavity structures, (**b**) Topological cavity structure, (**c**) Normalized electric field along the length of structure at zero-degree incidence angle, and (**d**) Normalized electric field at varying analyte refractive index for topological cavity structure.

The angular dispersion analysis is carried out to see the impact of incidence angle on the resonance mode excitation characteristics of both FP-cavity and topological cavity structures. Increasing the incidence angle leads to the excitation of high energy resonance mode for both configurations. Figure 5a represents the impact of the incidence angle on the resonance mode excitation characteristics for FP-cavity structures. In contrast, the same for topological cavity configuration is demonstrated in Fig. 5b. The structure shows mode excitation at 1578 nm, 1564 nm, 1521 nm, 1455 nm, and 1392 nm for FP-cavity structures at corresponding incidence angles of 0°, 10°, 20°, 30°, and 40°, respectively. Similarly, the structure shows mode excitation at 1659 nm, 1639 nm, 1579 nm, 1483 nm, and 1394 nm operating wavelengths for topological-cavity structures at corresponding incidence angles of 0°, 10°, 20°, 30°, and 40°, respectively. Additionally, both structures show a very narrow FWHM of less than 0.5 nm, thus showing its potential in tunable sensing applications. The angular-dependent response has been summarized in Table 1. The structure also possesses very high electric field confinement at the interface, as shown in Fig. 5c. The topological cavity shows a confined resonant mode electric field intensity of about 1.8176 × 10^6^, which is around 86% higher than the conventional FP-cavity structure.

**Table 1:** Impact of incidence angle on resonance mode excitation for both FP-cavity and topological cavity structures.

Incidence ANGLE (degree)	FP cavity		Topological cavity	
	Resonance wavelength (λ_r_) (nm)	FWHM (nm)	Resonance wavelength (λ_r_) (nm)	FWHM (nm)
0	1578.95	0.090	1659.23	0.059
10	1564.46	0.089	1639.33	0.055
20	1521.91	0.104	1579.73	0.051
30	1455.64	0.150	1483.54	0.055
40	1392.93	0.30	1394.01	0.40

Higher E-field intensity in the cavity improves light-matter interaction, resulting in sensitivity improvement. Moreover, the E-field intensity in the topological cavity structure is almost constant at various infiltrated analyte concentrations, as shown in Fig. 5d. This demonstrates the robust nature of the excited topological resonance mode.

The device sensor capability is investigated by considering a varying refractive index of the defect layer from 1.30 to 1.60. Infiltration of the analyte changes the effective refractive index of the aqueous defect layer, resulting in a shift in the resonance wavelength. The change in resonance wavelength ($\Delta \lambda_{r}$) is measured to calculate the concentration of the infiltrated analyte. This gives an average sensitivity ($S = \Delta \lambda_{r} /\Delta n$) of the devices, where $\Delta n$ is the refractive index variation having unit RIU^35^. Additionally, the performance of the proposed cavity structures is also characterized by calculating the quality factor (Q), and the figure of merit (FOM). Since the resonance peaks possess narrower FWHM (the bandwidth between the half point of the maximum reflectance spectrum), which gives the quality factor ($Q = \lambda_{r} /FWHM$)^36^, and figure of merit ($FOM = S/FWHM$)^37^. The impact of defect layer thickness on sensing performance of both the considered structures has been comparatively evaluated.

**Figure 6:**
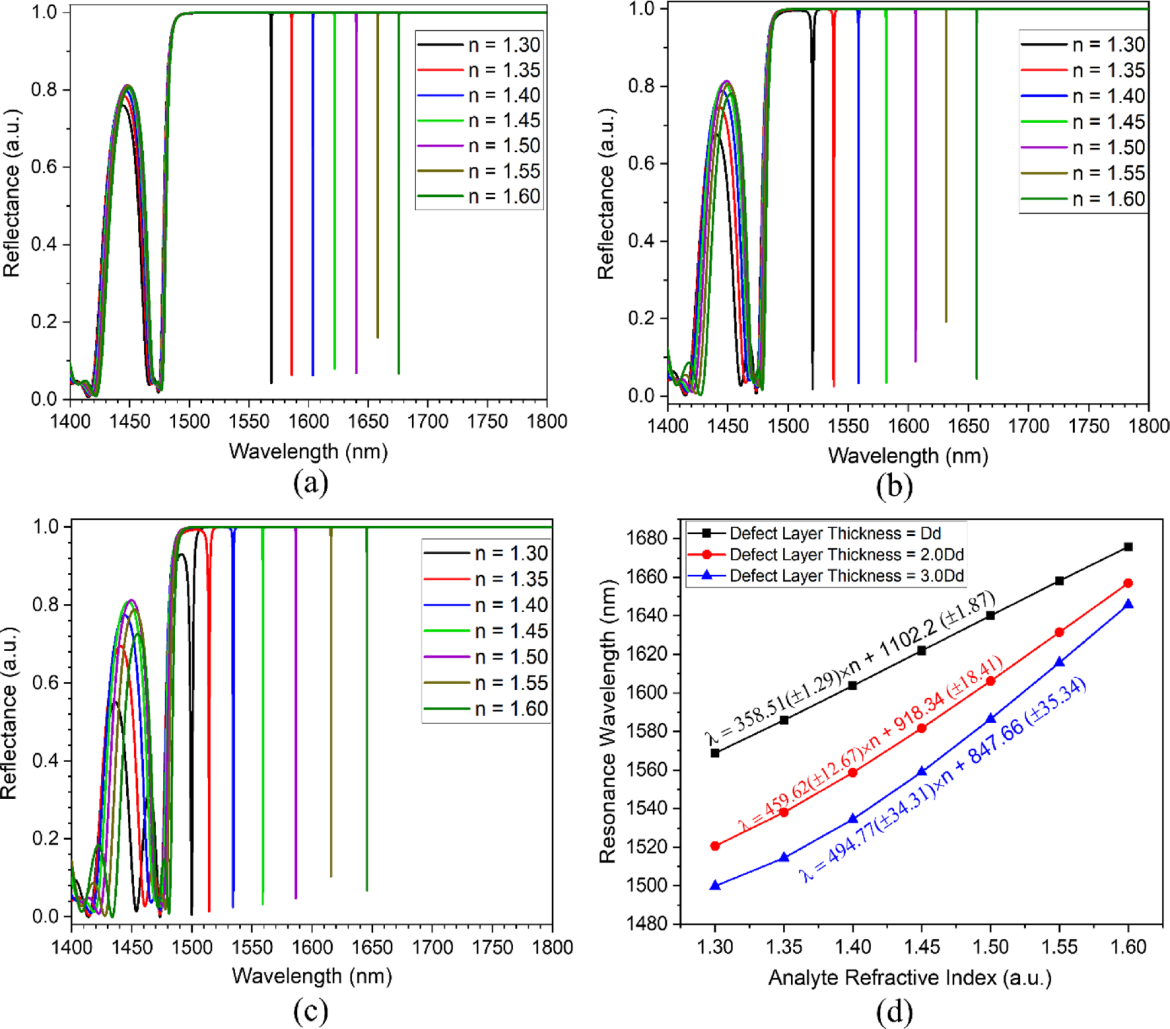
Sensitivity analysis for FP cavity (**a**) Defect layer thickness Dd, (**b**) Defect layer thickness 2Dd, (**c**) Defect layer thickness 3Dd, and (**d**) sensitivity summary.

The sensitivity response of the proposed FP-cavity structure at varying defect layer thicknesses is represented in Fig. 6. Increasing the defect layer thickness from Dd to 3Dd leads to the excitation of higher energy modes of improved sensitivity. Figure 6a–c represents the reflectance response of 1D-PhC-based FP-cavity heterostructure having corresponding defect layer thicknesses of 500 nm, 1000 nm, and 1500 nm, respectively. The infiltration of the analyte leads to an increase in the effective index of the defect layer, which results in a redshift of cavity resonance wavelength. The infiltrated analyte refractive index (n) dependent shift in resonance wavelength for different defect layer thicknesses can be calculated by Eqs. (2–4). This gives an average sensitivity of around 353 nm/RIU, 410 nm/RIU, and 402 nm/RIU for the corresponding defect layer thicknesses of Dd (500 nm), 2Dd (1000 nm), and 3Dd (1500 nm), respectively. The comparative results have been demonstrated in Fig. 6d, and structural performance parameters have been summarized in Table 2 for 1D-PhC-based FP-cavity structure.

2 $$\lambda_{Dd}^{FP} = 358.51\left( { \pm 1.29} \right) \times n + 1102 \left( { \pm 1.87} \right)$$

3 $$\lambda_{2Dd}^{FP} = 459.62\left( { \pm 12.67} \right) \times n + 918.34 \left( { \pm 18.41} \right)$$

4 $$\lambda_{3Dd}^{FP} = 494.77\left( { \pm 34.31} \right) \times n + 847.66 \left( { \pm 35.34} \right)$$

**Table 2:** Sensing performance summary of the conventional FP-cavity at various defect layer thicknesses.

Cavity width	n	Resonance wavelength (nm)	FWHM (nm)	Q	Sensitivity (nm/RIU)	FOM (RIU^−1^)
Dd	1.30	1568.77	0.110	14261.55	–	–
	1.35	1585.92	0.090	17621.33	343.0	3811.11
	1.40	1603.72	0.055	29158.55	349.5	6354.55
	1.45	1621.88	0.042	38616.19	354.1	8430.16
	1.50	1640.10	0.040	41002.50	356.7	8916.25
	1.55	1658.13	0.033	50246.36	357.4	10831.52
	1.60	1675.81	0.031	54058.39	356.8	11509.68
2Dd	1.30	1520.59	0.620	2452.57	–	–
	1.35	1538.04	0.260	5915.54	349.0	1342.31
	1.40	1558.70	0.125	12469.60	381.1	3048.80
	1.45	1581.68	0.070	22595.43	407.3	5818.10
	1.50	1606.15	0.040	40153.75	427.8	10695.00
	1.55	1631.45	0.030	54381.67	443.4	14781.33
	1.60	1656.99	0.024	69041.25	454.7	18944.44
3Dd	1.30	1499.79	1.950	769.12	–	–
	1.35	1514.38	0.760	1992.61	291.8	383.95
	1.40	1534.46	0.275	5579.85	346.7	1260.73
	1.45	1559.00	0.105	14847.62	394.8	3759.37
	1.50	1586.51	0.052	30509.81	433.6	8338.46
	1.55	1615.73	0.030	53857.617	463.8	15458.67
	1.60	1645.77	0.022	74807.73	486.6	22118.18

The FP-cavity of width 3Dd shows an average high-quality factor of around 2.6 × 10^4^, FWHM of 0.45 nm, and a maximum average sensitivity of about 403 nm/RIU, which gives the average FOM of about 8553 RIU^−1^. The obtained average FWHM, sensitivity, and FOM values are in comparison with various recently reported values. However, these can further be improved by considering topological 1D-PhC heterostructure cavities.

**Figure 7:**
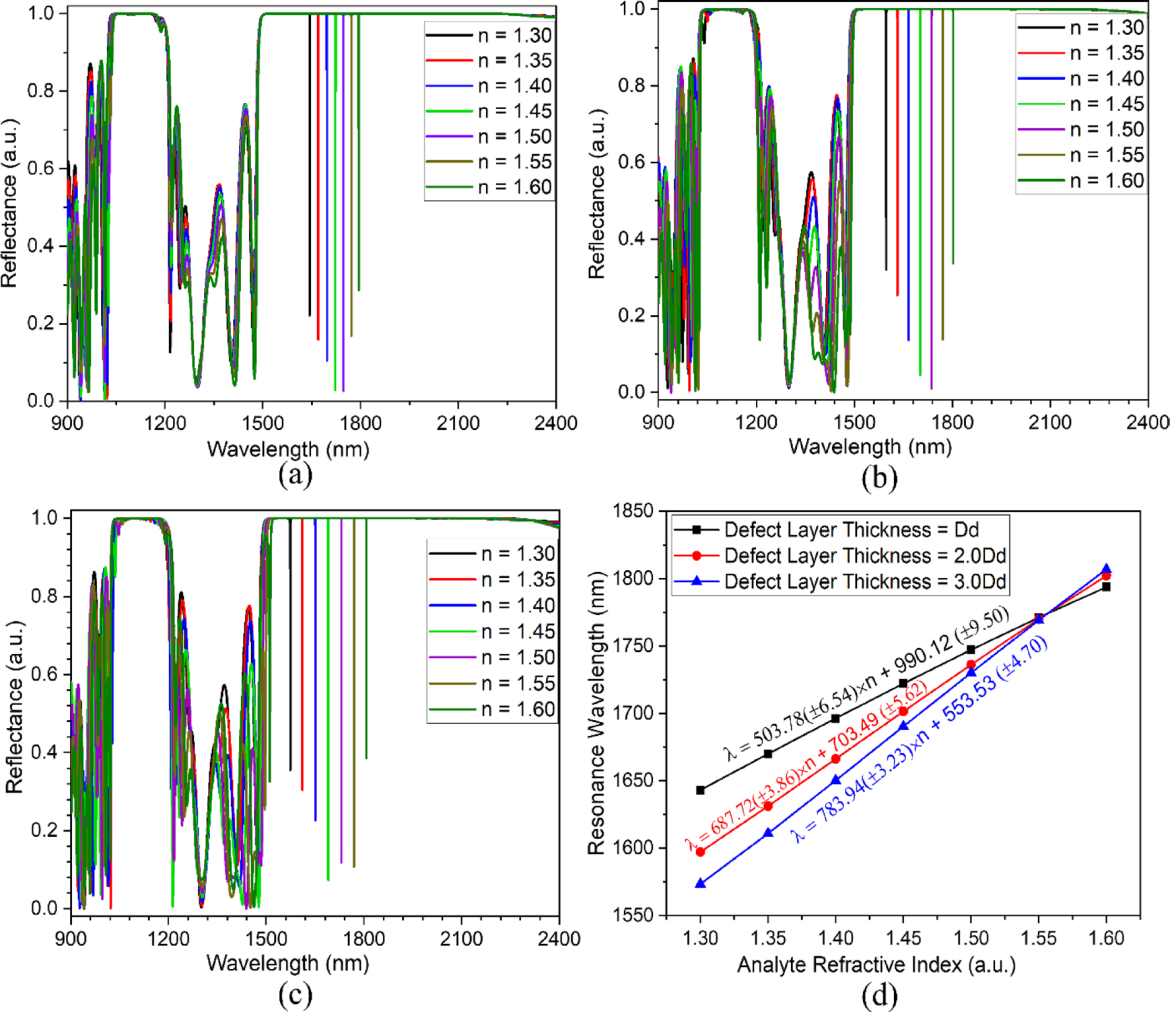
Sensitivity analysis for Topological cavity (**a**) Defect layer thickness Dd, (**b**) Defect layer thickness 2Dd, (**c**) Defect layer thickness 3Dd, and (**d**) sensitivity summary.

The sensitivity response of the proposed topological cavity structure at varying defect layer thickness is shown in Fig. 7. The higher energy modes are again excited for an increasing defect layer thickness (Dd–3Dd). It is noteworthy that the topological resonance mode is only excited for the second overlapped PBG because of their different topological properties. Figure 7a–c represents the reflectance response of the topological cavity heterostructure for the defect layer thicknesses of 570 nm, 1040 nm, and 1710 nm, respectively. The infiltration of the analyte leads to a red shift in resonance wavelength. This gives an average sensitivity of around 503 nm/RIU, 687 nm/RIU, and 783 nm/RIU for the corresponding defect layer thicknesses of Dd (570 nm), 2Dd (1040 nm), and 3Dd (1710 nm), respectively. The comparative results have been demonstrated in Fig. 7d, and structural performance parameters have been summarized in Table 3 for topological cavity structure.

**Table 3:** Sensing performance summary of the topological cavity at various defect layer thicknesses.

Cavity width	n	Resonance wavelength (nm)	FWHM (nm)	Q	Sensitivity (nm/RIU)	FOM (RIU^−1^)
Dd	1.30	1643.18	0.067	24525.07	–	–
	1.35	1670.00	0.055	30363.64	536.40	9752.73
	1.40	1696.41	0.051	33262.94	532.30	10437.25
	1.45	1722.28	0.049	35148.57	527.33	10761.90
	1.50	1747.29	0.048	36401.88	520.55	10844.79
	1.55	1771.20	0.048	36900.00	512.08	10668.33
	1.60	1793.85	0.047	38167.02	502.23	10685.82
2Dd	1.30	1597.2	0.084	19014.29	–	–
	1.35	1631.24	0.054	30208.15	680.80	12607.41
	1.40	1666.31	0.041	40641.71	691.10	16856.09
	1.45	1701.52	0.036	47264.44	695.47	19318.52
	1.50	1736.24	0.035	49606.86	695.20	19862.86
	1.55	1770.00	0.035	50571.43	691.20	19748.57
	1.60	1802.32	0.031	58139.35	683.73	22055.91
3Dd	1.30	1573.43	0.115	13682.00	–	–
	1.35	1610.92	0.055	29289.45	749.80	13632.73
	1.40	1650.34	0.036	45842.78	769.10	21363.89
	1.45	1690.38	0.028	60370.71	779.67	27845.23
	1.50	1730.23	0.027	64082.59	784.00	29037.04
	1.55	1769.30	0.026	68050.00	783.48	30133.85
	1.60	1807.05	0.022	82138.64	778.73	35396.97

Figure 7d summarizes the resonance wavelength variation of the proposed structure, which can be calculated for different defect layer thicknesses by Eqs. (5–7).

5 $$\lambda_{Dd}^{TP} = 503.78\left( { \pm 6.54} \right) \times n + 990.12 \left( { \pm 9.50} \right)$$

6 $$\lambda_{2Dd}^{TP} = 687.72\left( { \pm 3.86} \right) \times n + 703.49 \left( { \pm 5.62} \right)$$

7 $$\lambda_{3Dd}^{FP} = 783.94\left( { \pm 3.23} \right) \times n + 553.53 \left( { \pm 4.70} \right)$$

It is evident from Fig. 7d that the infiltration of the analyte within the aqueous defect layer exhibits an excellent linear dependency. The analysis rivals that increasing the analyte refractive index leads to a decrease in the FWHM, resulting in an improvement in the quality factor and FOM. The topological cavity structure of width 3Dd shows a high-quality factor of around 5.2 × 10^4^, FWHM of 0.044 nm, and a maximum sensitivity of about 774 nm/RIU, which gives the FOM of about 26,234 RIU^−1^.

**Figure 8:**
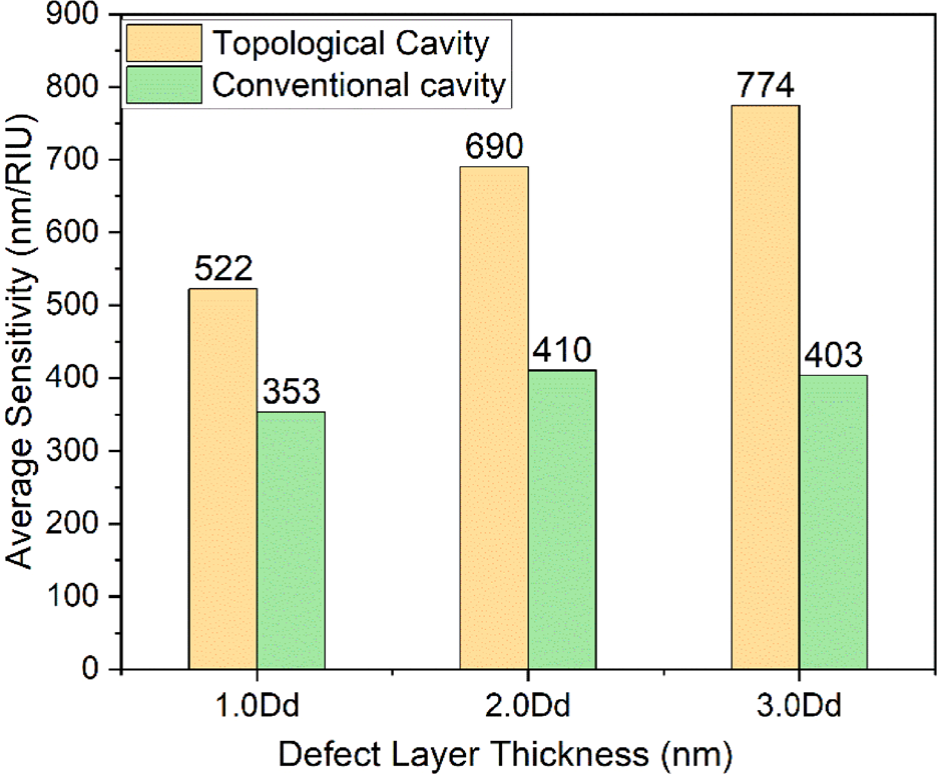
Comparative analysis of FP cavity and topological cavity structure.

The obtained average FWHM, sensitivity, and FOM values are much higher than various other recently reported values^38–40^. Therefore, the proposed structure exhibits its potential application as a refractive index-based optical sensor. Moreover, the considered refractive index range is wide enough to cover most biological and aqueous samples and thus is suitable for glucose sensing, hemoglobin measurement, and cholesterol sensing applications^41–43^. The comparative results of both 1D-PhC-based FP-cavity and topological heterostructures are shown in Fig. 8.

The topological heterostructures structure shows a 47%, 68%, and 92% improvement in sensitivity to conventional FP-cavity structure at corresponding defect layer thicknesses of Dd, 2Dd, and 3Dd, respectively. Finally, the structural performance has been compared with the recently reported values in Table 4.

**Table 4:** Comparative performance analysis with recently reported results.

Reference	Average sensitivity (nm/RIU)	Quality factor	FOM (RIU^−1^)	Year
^44^	638	25–30	471.9	2018
^45^	739	1.256 × 10^3^	737	2019
^46^	546.72	2.066 × 10^3^	–	2020
^47^	137.02	1.2 × 10^3^	200–700	2021
^48^	214.28	622.64	–	2023
^49^	496	1.2 × 10^5^	2.2 × 10^4^	2023
Proposed	774	5.2 × 10^4^	2.6234 × 10^4^	

## Conclusion

In this manuscript, a comparative performance analysis of the topological cavity and the conventional FP-cavity nanophotonic sensor structure is carried out. The structural parameters are optimized to tailor the dispersion characteristics, which shows a robust topological interface state excitation at a 1737 nm operating wavelength. The impact of cavity width on sensitivity is carried out, and detailed electric field confinement at the interface is studied. The analytical results demonstrate that the topological cavity structure exhibits superior performance than the conventional FP-cavity structure. The TPhC-cavity structure shows a 92% higher sensitivity, 98% improved Quality factor, 206% improvement in FOM, and 86% higher interface field confinement than conventional FP-resonator structures. Moreover, the structure possesses a broad sensing ability (RI: 1-3-1.6), thus showing its potential applications in biochemical sensors. Additionally, the low-cost, simple fabrication and characterization process makes it suitable for developing commercially highly sensitive planner nanophotonic devices.

## Data Availability

Data underlying the results presented in this paper are not publicly available at this time but may be obtained from the corresponding author upon reasonable request.
